# Angiotensin II Receptor Blocker Irbesartan Enhanced SIRT1 longevity Signaling Replaces the Mitochondrial Biogenetic Survival Pathway to Attenuate Hypertension-Induced Heart Apoptosis

**DOI:** 10.3390/jcdd9080266

**Published:** 2022-08-14

**Authors:** Pei-Ying Pai, James K. S. Wong, Zhen-Yang Cui, Yi-Yuan Lin, Shin-Da Lee

**Affiliations:** 1 Division of Cardiology, Department of Internal Medicine, China Medical University and Hospital, Taichung 40402, Taiwan; 2 Department of Cardiology, Asia University Hospital, Taichung 41354, Taiwan; 3 Department of Bioinformatics and Medical Engineering, Asia University, Taichung 41354, Taiwan; 4 School of Rehabilitation Medicine, Weifang Medical University, Weifang 261053, China; 5 Department of Exercise and Health Science, National Taipei University of Nursing and Health Sciences, Taipei City 11219, Taiwan; 6 Department of Physical Therapy, Graduate Institute of Rehabilitation Science, China Medical University, Taichung 406040, Taiwan; 7 Department of Physical Therapy, Asia University, Taichung 41354, Taiwan

**Keywords:** hypertension, heart, ARBs, peroxisome proliferator-activated receptor-γ, cell death, caspase

## Abstract

Background: The present study investigated whether angiotensin II type 1 receptor blocker irbesartan (ARB) and partial agonist of PPAR-γ prevents heart apoptosis by suppressing cardiac Fas/FasL-mediated to mitochondria-mediated apoptosis in the hearts of hypertensive rat model. Methods: Cardiac function using echocardiography, H&E staining, TUNEL assay, and Western blotting were measured in the excised hearts from three groups, i.e., an untreated hypertensive group (SHR), an ARB-treated hypertensive group (50 mg/kg/day, S.C., SHR-ARB), and untreated normotensive Wistar-Kyoto rats (WKY). Results: Fas Ligand, Fas death receptors, FADD, active caspase-8, active caspase-3 (Fas/FasL-mediated apoptotic pathway), as well as Bax, cytochrome c, active caspase-9 and -3 (mitochondria-mediated apoptotic pathway), IGF-II, and *p*-JNK were decreased in SHR-ARB group when compared with the SHR group. SIRT1, PGC-1α, Bcl2, and Bcl-xL (SIRT1/PGC-1α pro-survival pathway) were increased in the SHR-ARB group when compared with the SHR group. Conclusions: Our findings suggested that the ARB might prevent cardiac Fas/FasL-mediated to mitochondria-mediated apoptosis pathway in the hypertensive model associated with IGF-II, *p*-JNK deactivation, and SIRT1/PGC-1α pro-survival pathway upregulation. ARB prevents hypertension-enhanced cardiac apoptosis via enhancing SIRT1 longevity signaling and enhances the mitochondrial biogenetic survival pathway.

## 1. Introduction

Sustained hypertension, hypertension-induced myocardial morphology, and hypertension-induced myocardial dysfunction were associated with hypertrophic pathology, interstitial fibrosis, or cardiomyocyte apoptosis [1,2]. Therefore, it has great clinical significance to eliminate hypertension-induced myocardial cellular damage for preventing and treating pathological changes in hypertensive patients.

Angiotensin II was reported to activate downstream signaling that elicits various biological responses by binding the angiotensin type 1 (AT_1_R) receptor [3]. A previous study showed that angiotensin II stimulated the expression of Insulin-like growth factor II (IGF-II) and IGF-II receptors, which are mediated by mitogen-activated protein kinase kinase (MEK)/c-Jun N-terminal kinase (JNK) pathways in angiotensin II-stimulated H9c2 cardiomyoblasts and in rat hearts with abdominal aorta ligation [4,5]. Then, IGF-II somehow activated cellular death programs via the extrinsic Fas death receptor-dependent and intrinsic mitochondria-dependent apoptotic pathways [4]. When the Fas ligand binds the Fas death receptors on the cell membrane, the formation of a death-inducing signaling complex can activate the Fas-associated death domain (FADD), which in turn recruits the downstream caspase-8, enhances caspase-3 cleavage, and activates the cell death program [6]. The activation of the mitochondrial pro-apoptotic molecule Bcl-2-associated X protein (Bax) involves the apoptotic progression through enhanced cytochrome c release from the mitochondria into the cytosol, resulting in the cleavage of downstream effector caspase-9 and caspase-3 [4,7]. Previous studies indicated that the Fas to mitochondrial-dependent apoptotic pathways were activated in hypertension [8]. Moreover, apoptotic activity has been reported to contribute to the loss of cardiac myocytes and is recognized as an important predictor of adverse outcomes in patients with atherosclerotic cardiovascular disease [9]. The excessive accumulation of apoptosis, in turn, further attenuates bioenergetics efficiency and biogenesis of mitochondria, which is considered to be a key event in the development of cardiac pathologies [10]. Similarly, tissue from animals and humans with heart failure has reduced activity of the transcription factor responsible for mitochondrial biogenesis, PPAR-γ co-activator-1α (PGC-1α) [11]. Furthermore, the activation of the SIRT1/PGC-1α pathway was reported to play a protective role during mitochondrial-dependent apoptosis [12].

Compared with other AT_1_R antagonists, such as Losartan and Olmesartan, Irbesartan (ARB) has a potent and highly selective AT_1_R blocker that enables it to partially activate peroxisome proliferator-activated receptor gamma (PPAR-γ) agonistic effects, and has been approved worldwide for the treatment of hypertension in animals and humans [13,14,15]. The long-lasting, effective effect of ARB has been confirmed based on the binding of AT_1_R and slowly-dissociating AT_1_R antagonists [13]. Previous studies demonstrated that an ARB treatment provides protective cardiovascular effects beyond its antihypertensive action [16,17,18]. Our previous study reported that ARB might inhibit chronic intermittent hypoxia-enhanced cardiac apoptosis via JNK deactivation and SIRT1 upregulation [19]. However, whether an ARB treatment could be beneficial to hypertension-induced widely dispersed apoptosis in the heart is still unclear. In the study, we hypothesized that ARB might prevent cardiac Fas/FasL-mediated to mitochondria-mediated apoptotic pathways and enhance the cardiac SIRT1/PGC-1α pro-survival pathway in hypertension.

## 2. Materials and Methods

### 2.1. Animal Model

A total of 28 8-weeks-old male spontaneously hypertensive rats and 14 8-weeks-old male Wistar Kyoto rats (WKY) were fed a standard laboratory chow (Lab Diet 5001; PMI Nutrition International Inc., Brentwood, MO, USA) and water ad libitum, the ambient temperature was maintained at 25 °C and maintained on an artificial 12-h light–dark cycle. The spontaneously hypertensive rats were divided into ARB (50 mg/kg, S.C. per day, SHR-ARB) groups and vehicle (0.5% methylcellulose, SHR) groups. The normotensive WKY rats that were administered the vehicle (0.5% methylcellulose, WKY) treatment served as a control. The dose of ARB was set at 50 mg/kg/day to correspond to the dose of ARB used in humans [19,20]. The rats received a daily subcutaneous injection of either ARB or vehicle for 8 weeks.

### 2.2. Tail Cuff Resting Blood Pressure and Echocardiography

The blood pressure was determined in conscious rats by a tail-cuff method (LE5001, Panlab, Wood Dale, IL, USA). The following parameters were measured: resting heart rate and blood pressure (systolic blood pressure, diastolic blood pressure, and mean blood pressure). The echocardiographic evaluations of rats were obtained using a commercially available transthoracic echocardiography system (M2424A Philips ultrasound systems, Andover, MA, USA) with a 10 MHz linear transducer, as previously described [8].

### 2.3. Histological Analysis

The heart tissue samples (6 rat hearts in each group) were fixed with 4% paraformaldehyde and then embedded in paraffin. The slides were stained with hematoxylin–eosin (Merck, Darmstadt, Germany) following standard procedures. Photomicrographs of the heart sections were obtained in at least 6 separate fields × 2 slides × 3 left ventricle regions per condition (6 rat hearts in each group) for data quantification.

### 2.4. TUNEL Assay in Heart Tissue

Cardiac apoptotic nuclei were performed with a Situ Cell Death Detection Kit (Roche, Indianapolis, IN, USA) assay according to the manufacturer’s instructions. In brief, the sections were treated with proteinase K, followed by permeabilization solution, a blocking buffer. A TUNEL and fluorescein isothiocyanate-dUTP assay proceeded for 60 min at 37 °C. Finally, all of the slides were mounted using a vectashield with 4,6-diamidino-2-phenylindole (DAPI). The number of TUNEL-positive cardiomyocytes was examined using a fluorescent microscope (DP 74, Olympus, Tokyo, Japan).

### 2.5. Immunoblot Analysis

The total protein from the left ventricle tissue was homogenized using a lysis buffer at a ratio of 100 mg tissue/1 mL buffer. The supernatant was collected, and the protein concentration was measured using the Bradford method (Bio–Rad Laboratories, Hercules, CA, USA). The membranes were incubated overnight at 4 °C with primary antibodies, including Fas Ligand, Fas, FADD, Bax, cytosolic cytochrome c, active caspase-8, active caspase-9, and active caspase-3, IGF-II, *p*-JNK, SIRT1, PGC-1, Bcl-2, Bcl-xL, and α-tubulin (Santa Cruz Biotechnology, Santa Cruz, CA, USA). The membranes were washed and incubated with secondary antibodies, including goat anti-rabbit IgG-HRP, goat anti-mouse IgG-HRP, or goat anti-donkey IgG-HRP (Santa Cruz). The blots were visualized with an enhanced chemiluminescence ECL kit (Millipore Corporation, Billerica, MA, USA). Densitometric analysis was performed using a bioimaging analyzer (LAS-3000; Fujifilm Corporation, Tokyo, Japan).

### 2.6. Statistical Analysis

The data are presented as the means ± standard deviation (SD). The significant differences among groups were determined by a one-way analysis of variance followed by Tukey’s multiple comparison test. A value of *p* < 0.05 was considered statistically significant.

## 3. Results

### 3.1. Body Weight and Cardiac Characteristics 

**Figure 1:**
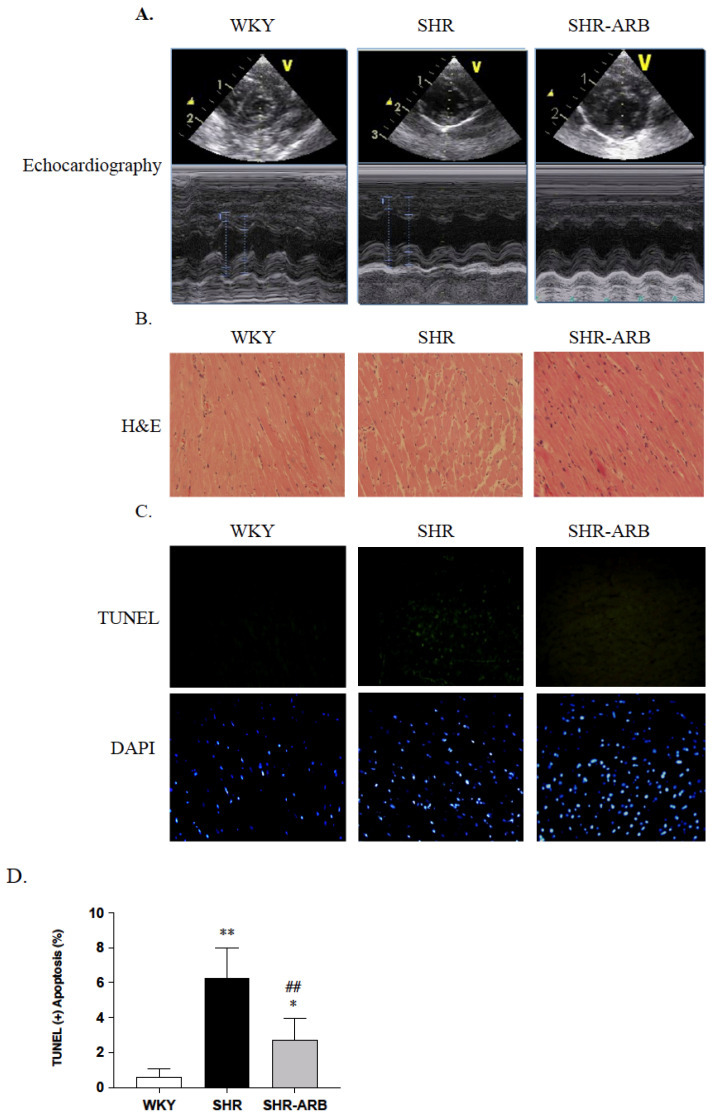
(**A**) Representative M-mode echocardiographic parameters were recorded through the anterior and posterior left ventricular walls and obtained with a 2D short-axis view at the level of the papillary muscles. (**B**) representative H&E (hematoxylin and eosin stain). (**C**) representative TUNEL (terminal deoxynucleotide transferase-mediated dUTP nick end labeling) assay (top: green spots) and DAPI staining (bottom: blue spots) of cardiac sections from left ventricles in the normotensive group (WKY), untreated hypertensive group (SHR), and SHR rats with treated irbesartan (SHR-ARB). The images were magnified ×400. (**D**) Bars indicate the percentage of TUNEL-positive cells and show mean ± standard deviation, (n = 6;6;6). * *p* < 0.05, ** *p* < 0.001: significant differences from the WKY group. * *p* < 0.05 significant differences from the Control group. ## *p* < 0.01 indicates significant differences between the SHR group and the SHR-ARB group.

The whole heart weight (WHW), left ventricular weight (LVW), WHW/body weight (BW), LVW/BW, LVW/WHW, WHW/tibia length (TL), and LVW/TL were significantly reduced in the SHR-ARB groups, when compared with the untreated SHR group (Table 1). Echocardiography revealed significantly reduced left ventricular wall thickness in the interventricular septum at diastole (IVSd) and internal dimension at the systole of the left ventricle (LVIDs), as well as significantly increased Fractional Shortening (FS) in the SHR-ARB groups when compared with untreated SHR group (Table 1 and Figure 1A). The systolic blood pressure, diastolic blood pressure, and mean blood pressure were significantly reduced in the SHR-ARB groups when compared with the untreated SHR group (Table 1). In addition, by viewing the 400× magnified images, increased ventricular interstitial spaces were found in the SHR group compared with the WKY group. The myocardial architecture abnormalities in the SHR-ARB group were less than those in the untreated SHR group (Figure 1B).

### 3.2. DAPI Staining and TUNEL-Positive Apoptotic Cells of Left Ventricle

To analyze the anti-apoptotic effect of ARB in hypertension, DAPI staining and a TUNEL assay were used on the WKY, SHR, and SHR-ARB groups. We observed that the left ventricle of the SHR group had a greater percentage of TUNEL-positive cardiac cells than the left ventricles of the WKY group. In contrast, the percentage of the TUNEL-positive cardiac cells in the SHR-ARB group was lower than that in the untreated SHR group (Figure 1C,D)

### 3.3. Upstream of Fas/FasL- to Mitochondria-Mediated Apoptotic Pathways

**Figure 2:**
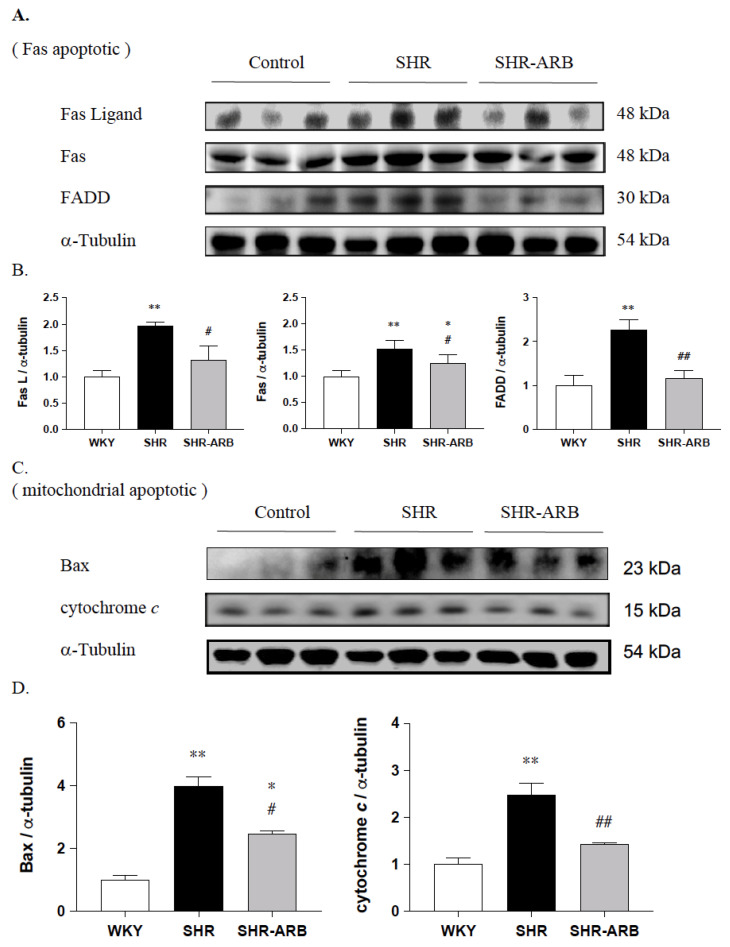
(**A**) The representative protein levels of Fas ligand, Fas receptor (Fas), and Fas-associated death domain (FADD). The α-Tubulin served as an internal control. (**B**) Bars indicate the relative fold changes of protein levels relative to the Control group in Fas ligand, Fas, and FADD on α-Tubulin and mean ± standard deviation (n = 8;8;8). (**C**) The representative protein levels of pro-apoptotic Bax and cytosolic cytochrome *c* from the left ventricles in the normotensive group (WKY), untreated hypertensive group (SHR), and SHR rats with treated irbesartan (SHR-ARB), as measured by Western blot methods. The α-Tubulin served as an internal control. (**D**) Bars indicate the relative fold changes of protein levels relative to the Control group in Bax and cytosolic cytochrome *c* on α-Tubulin and show mean ± standard deviation (n = 8;8;8). * *p* < 0.05, ** *p* < 0.01: significant differences from the Control group. # *p* < 0.05, ## *p* < 0.01 indicates significant differences between the SHR group and the SHR-ARB group.

We further determined the expression of the upstream cardiac Fas/FasL-to-mitochondria-mediated apoptotic pathways in hypertension; the protein levels of Fas ligand, Fas, and FADD, as well as Bax and cytochrome *c*, were measured by Western blot methods in the left ventricle excised from the WKY, SHR, and SHR-ARB groups. When compared with the WKY group, the protein levels of Fas ligand, Fas, and FADD, as well as Bax and cytochrome *c*, were significantly increased in the SHR group (Figure 2). The protein levels of Fas ligand, Fas, and FADD, as well as Bax and cytochrome *c*, in the SHR-ARB group were significantly lower than that in the untreated SHR group (Figure 2).

### 3.4. Downstream of Fas/FasL- to Mitochondria-Mediated Apoptotic Pathways

**Figure 3:**
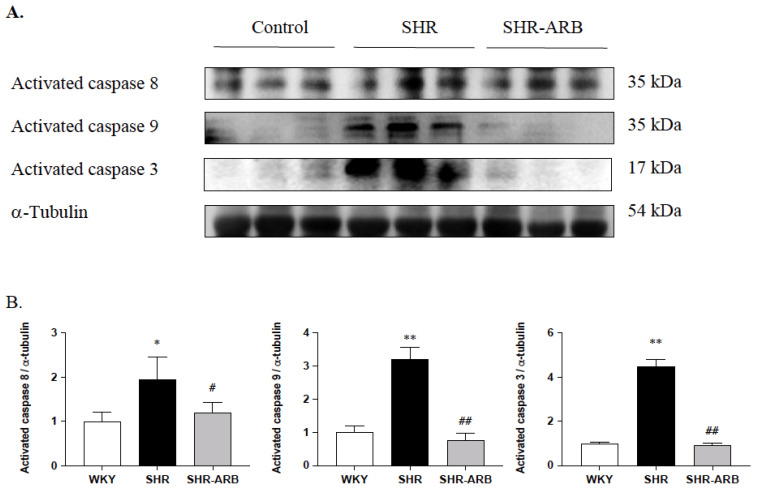
(**A**) The representative protein levels of caspase-8, caspase-9, and caspase-3 from the left ventricles in the normotensive group (WKY), untreated hypertensive group (SHR), and SHR rats with treated irbesartan (SHR-ARB), as measured by Western blot methods. α-Tubulin served as an internal control. (**B**) Bars indicate the relative fold changes of protein levels relative to the Control group in active caspase-8, caspase-9, and caspase-3 on α-Tubulin and mean ± standard deviation (n = 8;8;8). * *p* < 0.05, ** *p* < 0.01: significant differences from the Control group. # *p* < 0.05, ## *p* < 0.01 indicates significant differences between the SHR group and the SHR-ARB group.

We next measured the downstream cardiac Fas/FasL- and mitochondria-mediated apoptotic pathways, the active form of activated caspases-8, -9, and -3 were measured by Western blot methods in the left ventricle excised from the WKY, SHR, and SHR-ARB groups. The protein levels of activated caspases-8, -9, and -3 in untreated SHR groups were found to be elevated when compared to the WKY group (Figure 3). The protein levels of active caspases-8, -9, and -3 in the SHR-ARB group were significantly lower than that in the untreated SHR group (Figure 3).

#### 3.4.1. Effect of ARB on IGF-II and p-JNK Protein Expression

**Figure 4:**
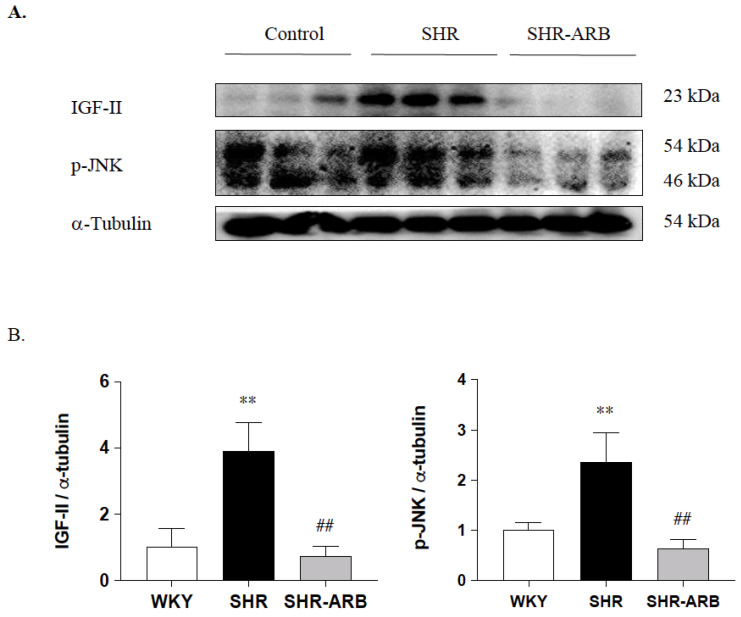
(**A**) The representative protein levels of IGF-II and *p*-JNK from the left ventricles in the normotensive group (WKY), untreated hypertensive group (SHR), and SHR rats with treated irbesartan (SHR-ARB), as measured by Western blot methods. The α-Tubulin served as an internal control. (**B**) Bars indicate the relative fold changes of protein levels relative to the Control group in IGF-II and *p*-JNK on α-Tubulin and show mean ± standard deviation (n = 8;8;8). ** *p* < 0.01: significant differences from the Control group. ## *p* < 0.01 indicates significant differences between the SHR group and the SHR-ARB group.

We further examined the effects of the ARB treatment on IGF-II and *p*-JNK in hypertension; the protein levels of IGF-II and *p*-JNK were measured by Western blot methods in the left ventricle excised from the WKY, SHR, and SHR-ARB groups. The protein levels of IGF-II and *p*-JNK were significantly increased in the untreated SHR group compared with the WKY group, whereas those were significantly decreased in the SHR-ARB group compared with the untreated SHR group (Figure 4).

#### 3.4.2. Cardiac SIRT1/PGC-1α Pro-Survival Pathway

**Figure 5:**
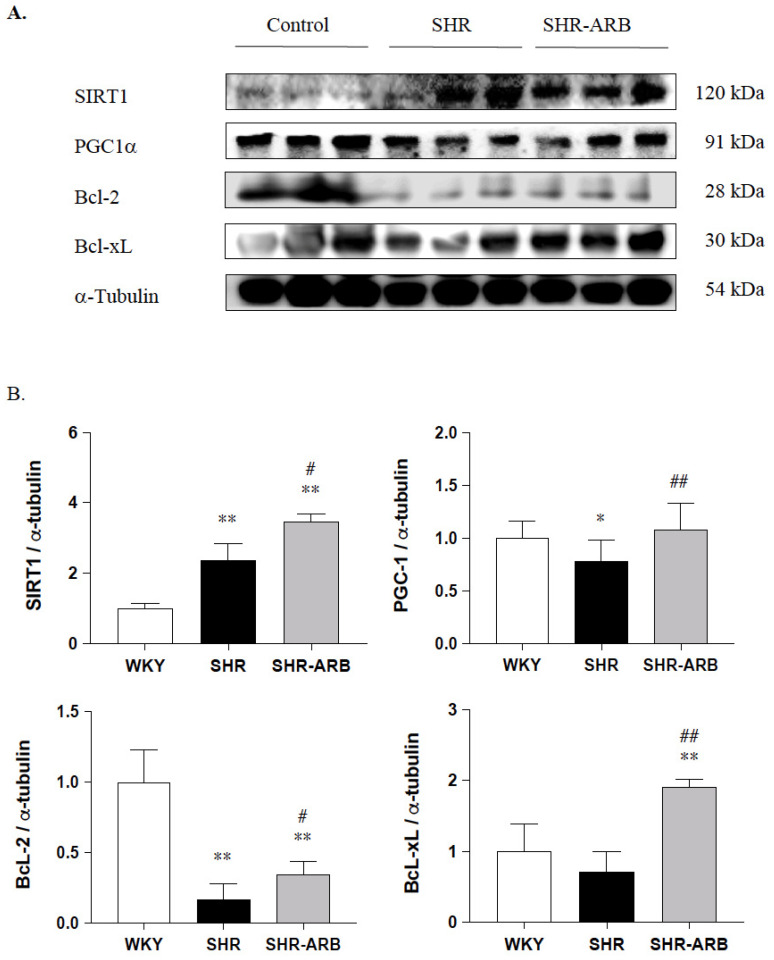
(**A**) The representative protein levels of SIRT1, PGC-1, BcL-2, and BcL-xL extracted from the left ventricles of excised hearts in the normotensive group (WKY), untreated hypertensive group (SHR), and SHR rats with treated irbesartan (SHR-ARB), as measured by Western blot methods. α-Tubulin served as an internal control. (**B**) Bars indicate the relative fold changes of protein levels relative to the Control group in SIRT1, PGC-1, BcL-2, and BcL-xL based on α-Tubulin and show mean ± standard deviation (n = 8;8;8). * *p* < 0.05, ** *p* < 0.01: significant differences from the Control group. # *p* < 0.05, ## *p* < 0.01 indicates significant differences between the SHR group and the SHR-ARB group.

Next, to evaluate the pro-survival effects of the ARB treatment on the cardiac SIRT1/PGC-1α pro-survival pathway in hypertension, the cardiac SIRT1/PGC-1α pro-survival relative protein expression was measured by Western blot methods in the left ventricles excised from the WKY, SHR, and SHR-ARB groups. The protein levels of cardiac SIRT1 were enhanced in the untreated SHR group compared with the WKY, as well as further increased in SHR-ARB when compared with the untreated SHR group (Figure 5). The pro-survival protein levels of PGC-1α and BcL-2, except BcL-xL, in the untreated SHR groups were significantly lower than those in the WKY group (Figure 5). The SIRT1/PGC-1α pro-survival protein levels of PGC-1α, BcL-2, and BcL-xL in the SHR-ARB group were significantly higher than those in the untreated SHR group (Figure 5).

## 4. Discussion

**Figure 6:**
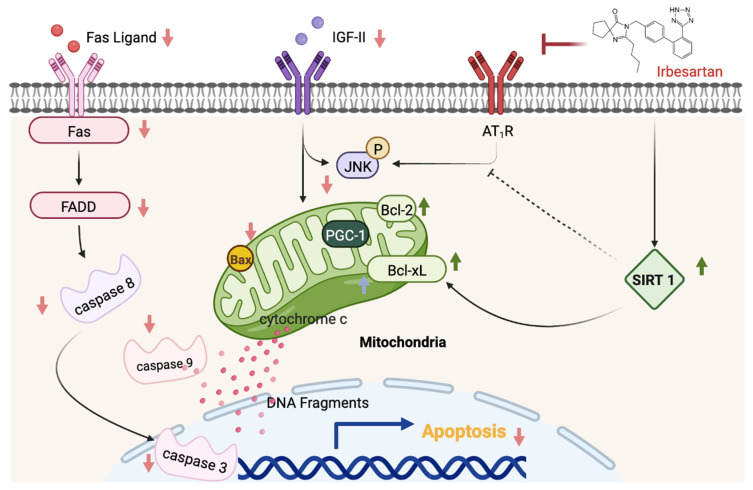
Hypothesized diagram. The proposed schematic diagram from the current study shows that the cardiac Fas/FasL-mediated (Fas Ligand, Fas, FADD, and active caspase-8) and the mitochondria-mediated apoptotic pathways (Bax, cytosolic cytochrome c, Active caspase-9, and Active caspase-3) were activated by hypertension through IGF-II and *p*-JNK activation. Moreover, the pro-survival protein of SIRT1 appeared to have a compensatory pro-survival response after hypertension. However, these cardiac Fas/FasL-mediated and mitochondria-mediated apoptotic pathways appear to be suppressed or not activated via the enhanced SIRT1/PGC-1α pro-survival pathway (SIRT1, PGC-1, Bcl-2, and Bcl-xL) under hypertension after treated Irbesartan.

Our key findings were as follows: (1) The ARB treatment decreased the myocardial architecture abnormalities and TUNEL-positive apoptotic cells in hypertension. (2) The ARB treatment attenuated the Fas/FasL-mediated apoptotic protein levels (Fas Ligand, Fas death receptors, FADD, active caspase-8), and mitochondria-mediated apoptotic protein levels (Bax, cytochrome c, active caspase-9, and active caspase-3) in hypertension. (3) Hypertension enhances the IGF-II and *p*-JNK expression, whereas the ARB treatment decreases cardiac IGF-II and *p*-JNK expression. (4) The ARB treatment enhanced the cardiac SIRT1 protein, as we as active SIRT1/PGC-1α pro-survival pathway (SIRT1, PGC-1α, Bcl2, and Bcl-xL) in hypertension. The current study supported our expected hypothesis that the ARB treatment might attenuate myocardial Fas/FasL- and mitochondria-mediated apoptotic pathways by decreasing IGF-II and *p*-JNK and enhancing the SIRT1/PGC-1α pro-survival pathway in hypertensive rats (Figure 6). Therefore, the angiotensin II receptor might play a pathophysiologic role, and AT_1_R antagonist ARB might play a therapeutic role in widely dispersed apoptosis in hypertension.

Several lines of evidence demonstrate that the long-lasting effect of ARB has been confirmed by its strong binding to AT_1_R and slow dissociating from AT_1_R compared with other Angiotensin-II-receptor blockers such as losartan and olmesartan [18,20,21]. Based on this viewpoint, the prevention of Angiotensin-II signaling by AT_1_R blockers ARBs has been extensively used in the therapy of hypertension. Watanabe et al., demonstrated the suppressive effects of ARB on left ventricular function decline, cardiac fibrosis, and hypertrophy through inflammation and oxidative stress in rats with myocardial infarction [18]. Similarly, Zhao et al., showed that ARB had potential therapeutic effects on atherosclerosis via anti-inflammatory and anti-apoptotic mechanisms [22]. The mechanism of ARB could be co-related to cardiovascular protection via AT_1_R/PPAR-γ activation. The results of our present study were in agreement with previous reports that ARB administration could prevent hypertension, enlarged interstitial space, higher IVSd, and lower fractional shortening. Moreover, the ARB treatment for hypertension preserved the normal morphology of the myocardium.

The Ang-II-induced apoptosis in myocardial cells was shown to be mediated by the AT_1_ receptor in hypertension [3]. Widely dispersed apoptosis in the heart is one of the major pathogenic mechanisms that cause heart failure [9]. Importantly, one study has indicated that high salt intake induced AT_1_ receptor upregulation and mediated IGF-II expressions to develop cardiac pathological hypertrophy [23]. The ability to block the apoptosis process could prevent or delay the loss of myocardial cells and minimize hypertension-induced myocardial injury. In this current study, the ARB treatment not only suppressed the Fas/FasL-mediated and mitochondria-mediated apoptotic pathways but also attenuated IGF-II expression. This implied that the ARB treatment could diminish the hypertension-activated Fas/FasL-mediated and mitochondria-mediated apoptotic pathways, possibly by decreasing IGF-II signaling. From the findings, we demonstrate that IGF-II plays a crucial role in high blood pressure leading to cardiac widely dispersed apoptosis, which might provide a new target in a therapeutic approach to cardiac disease.

PGC-1α has been found to be an essential regulator of mitochondrial biogenesis and plays a crucial role in the development of cardiovascular diseases [11,24,25]. Growing evidence has demonstrated that the PGC-1α target genes are downregulated in animal and human models of failing hearts [26,27]. In addition, our previous study indicated that the coexistence of hypertension and ovariectomy could additively promote the widely dispersed cardiac apoptosis-related death process associated with PGC-1α downregulation [28]. Similarly, we observed that cardiac PGC-1α protein levels were significantly downregulated in hypertension, whereas the ARB treatment appeared to enhance PGC-1α in hypertensive rats. Supportively, ARB exerts its cardioprotective effect not only through a potent and highly selective AT_1_R blocker effect but also in part by modulating the PPAR-γ and PGC-1α pathways [13]. In our study, the ARB blocking AT_1_R with a partial agonist of PPAR-γ treatment appeared to upregulate the PGC-1 signaling pathway, which provides supportive evidence that ARB plays an anti-apoptotic role in hypertensive hearts. It is well known that ARB could have cardioprotective effects, possibly due to PPAR-γ activation [15,29]. To the best of our knowledge, this is the first study to describe the cardioprotective effect of ARB through the PGC-1 pathway in hypertensive rats.

Changes in SIRT1 expression are critical in metabolic syndrome, cardiovascular diseases, cancer, and neurodegeneration. SIRT1 plays a pivotal cardioprotective role in mediating the cell death/survival process [30,31]. However, a recent study reported that diseases or cardiovascular system dysfunctions are associated with an increase in SIRT1 expression levels, associated with a compensatory or protective increase in SIRT1 expression to deal with the decline of the SIRT1 activity. [32]. The literature reported that the activity of SIRT1 protein is directly or indirectly controlled via the JNK1-SIRT1 link by accumulated oxidative stress. Alcendor et al., noted that the rate of SIRT1 overexpression had a two-sided action on the cardiovascular system [31]. Our recent study reported that chronic nocturnal intermittent hypoxia-enhanced cardiac apoptosis led to JNK activation and SIRT1 upregulation [19]. In the present study, we observed that hypertension-enhanced cardiac apoptosis led to high levels of SIRT1 and *p*-JNK, as well as low levels of PGC-1α in the hearts of untreated hypertensive rats, which might imply that the increase in the SIRT1 level may be a compensatory mechanism in hypertension.

PPAR-γ agonists possess potent anti-inflammatory, anti-oxidative stress, and anti-apoptosis actions and protect against the impairment of mitochondrial function by upregulating BcL-2 expression [33]. Moreover, SIRT1 activation stimulates mitochondrial biogenesis through the deacetylation and transcriptional activation of the PGC-1α pro-survival pathway. Of note, SIRT1 not only plays a key role in mitochondrial biogenesis control but also activates the pro-survival pathway. Our previous study suggested that ARB markedly reduces apoptosis in the hearts of chronic nocturnal intermittent hypoxia-enhanced cardiac apoptosis via JNK deactivation and SIRT1 upregulation [19]. Consistent with previous studies, we observed that the ARB treatment exhibited significant anti-apoptotic potential, as evidenced by the upregulation of the SIRT1/PGC-1α pro-survival pathway and the downregulation of Fas/FasL-mediated and mitochondria-mediated apoptotic pathways together with a reduction in the percentage of TUNEL-positive cells in hypertension, which might imply that ARB might be a possible therapeutic approach to prevent or suppress hypertension-induced widely dispersed apoptosis in the heart associated with enhancing pro-survival pathways and mitochondrial biogenesis.

IGF-II signaling is crucial to myocardial pathological development and remodeling, which subsequently lead to heart failure [4,5]. High expressions of IGF-II were observed in the hearts of the hypertension model with widely dispersed apoptosis through JNK-activated SIRT1 degradation [4]. In the present study, the ARB-treated hypertensive rats exhibited significantly milder apoptosis and lower IGF-II and *p*-JNK expression in the myocardium compared with untreated hypertension rats, suggesting that ARB may reduce Fas/FasL-mediated and mitochondria-mediated pathways activation to alleviate myocardial abnormalities in hypertensive rats by reducing the expression of IGF-II and *p*-JNK.

Despite the positive effects of the present ARB on anti-apoptosis, we should consider some limitations in the current experimental design. Although our data suggest that ARB has anti-apoptosis effects, we were not able to determine the specific mechanisms responsible for these effects (that is, AT_1_R, PPAR-γ agonism, both, or other unknown mechanisms). Recent work has highlighted the important role of mitochondrial fusion/fission dynamics in mitochondrial homeostasis, which contributes to cardiovascular health and disease [34,35]. However, the current study focused on mitochondria-mediated apoptosis. Therefore, whether SIRT1/PGC-1α signaling is involved in the regulatory effect of ARB on mitophagy or mitochondrial dynamics is unknown in the pathogenesis of hypertension-induced cardiac dysfunction. Further studies are required to evaluate these points. Oxidative stress has been considered to promote mitochondrial dysfunction and lead to cardiac damage, which is involved in the pathogenic mechanism of hypertension. These signaling pathways play important roles in counteracting hypertension. Thus, the potential mechanisms that influence ARB on ROS and antioxidant activity for these impairments in hypertensive rats need to be further clarified. Despite these limitations, we believe that this study has provided important new information for the understanding of the effects of ARB on myocardial apoptosis and myocardial abnormalities in hypertension.

## 5. Conclusions

In conclusion, we demonstrate that hypertension may enhance cardiac Fas/FasL-mediated and mitochondria-mediated apoptosis, which may impair cardiovascular function. The dual AT_1_R blockers/PPAR-γ agonist ARB could prevent apoptotic progression through the upregulation of the cardiac SIRT1/PGC-1α pro-survival pathway associated with IGF-II and *p*-JNK deactivation in hypertension. This study provides a possible mechanism by which treatment with ARB ameliorates myocardial apoptosis and myocardial abnormalities in hypertension.

## Figures and Tables

**Table 1:** Cardiac characteristics of the WKY, SHR, and SHR-ARB groups.

Parameters/Groups	WKY	SHR	SHR-ARB
Number of animals	8	8	8
BW (g)	345 ± 35.46	400.6 ± 32.08 *	406.1 ± 19.86 **
WHW (g)	1.54 ± 0.37	1.78 ± 0.19	1.45 ± 0.06 ##
LVW (g)	0.996 ± 0.26	1.42 ± 0.17 *	1.09 ± 0.05 ##
WHW/BW (×10^4^)	45.84 ± 8.76	45.94 ± 2.27	35.69 ± 7.34 * ##
LVW/BW (×10^4^)	29.49 ± 5.53	36.58 ± 2.24 *	26.93 ± 9.30 ##
LVW/WHW	0.645 ± 0.038	0.796 ± 0.012 **	0.754 ± 0.014 ** ##
WHW/TL (g/mm)	0.041 ± 0.009	0.046 ± 0.005	0.037 ± 0.001 ##
LVW/TL (g/mm)	0.027 ± 0.007	0.037 ± 0.004 *	0.028 ± 0.001 ##
IVSd (mm)	1.78 ± 0.15	2.26 ± 0.28 **	1.84 ± 0.24 ##
LVPWd (mm)	1.80 ± 0.33	1.92 ± 0.25	1.88 ± 0.23
LVIDd (mm)	7.74 ± 0.59	8.63 ± 0.80	8.04 ± 0.44
LVIDs (mm)	4.75 ± 0.73	5.73 ± 0.80 *	4.76 ± 0.65 ##
Fractional Shortening (FS), %	38.6 ± 8.2	33.8 ± 4.2	41.04 ± 5.66 ##
Heart rate	424 ± 14	423 ± 13	404 ± 11
Systolic blood pressure (mmHg)	137 ± 9.36	225 ± 11.30 **	160 ± 12.90 ##
Diastolic blood pressure (mmHg)	81 ± 14.04	155 ± 21.30 **	97 ± 15.90 * ##
Mean blood pressure (mmHg)	100 ± 10.40	178 ± 15.70 **	118 ± 12 ** ##

Values are mean ± SD amount the normotensive group (WKY), untreated hypertensive group (SHR), and SHR rats with treated irbesartan (SHR-ARB). BW: body weight; WHW: whole heart weight; LVW: left ventricular weight; TL: tibia length; IVSd: interventricular septum at diastole; LVPWd: left ventricular posterior wall thickness at diastole; LVIDd: internal dimension at diastole of left ventricle; LVIDs: internal dimension at the systole of the left ventricle; FS: (LVIDd − LVIDs)/LVIDd × 100. * *p* < 0.05, ** *p* < 0.01 Significant differences between the WKY and SHR or SHR-ARB group. **##** *p* < 0.01 Significant differences between SHR group and SHR-ARB group.

## Data Availability

All data collected in the manuscript are available upon request.

## References

[1] Muller-Brunotte R., Kahan T., Lopez B., Edner M., Gonzalez A., Diez J., Malmqvist K. (2007). Myocardial fibrosis and diastolic dysfunction in patients with hypertension: Results from the Swedish Irbesartan Left Ventricular Hypertrophy Investigation versus Atenolol (SILVHIA). J. Hypertens..

[2] Gonzalez A., Fortuno M.A., Querejeta R., Ravassa S., Lopez B., Lopez N., Diez J. (2003). Cardiomyocyte apoptosis in hypertensive cardiomyopathy. Cardiovasc. Res..

[3] Hunyady L., Catt K.J. (2006). Pleiotropic AT1 receptor signaling pathways mediating physiological and pathogenic actions of angiotensin II. Mol. Endocrinol..

[4] Lee S.D., Chu C.H., Huang E.J., Lu M.C., Liu J.Y., Liu C.J., Hsu H.H., Lin J.A., Kuo W.W., Huang C.Y. (2006). Roles of insulin-like growth factor II in cardiomyoblast apoptosis and in hypertensive rat heart with abdominal aorta ligation. Am. J. Physiol. Endocrinol. Metab..

[5] Huang C.Y., Kuo W.W., Yeh Y.L., Ho T.J., Lin J.Y., Lin D.Y., Chu C.H., Tsai F.J., Tsai C.H., Huang C.Y. (2014). ANG II promotes IGF-IIR expression and cardiomyocyte apoptosis by inhibiting HSF1 via JNK activation and SIRT1 degradation. Cell Death Differ..

[6] Bishopric N.H., Andreka P., Slepak T., Webster K.A. (2001). Molecular mechanisms of apoptosis in the cardiac myocyte. Curr. Opin. Pharmacol..

[7] Crow M.T., Mani K., Nam Y.J., Kitsis R.N. (2004). The mitochondrial death pathway and cardiac myocyte apoptosis. Circ. Res..

[8] Huang C.Y., Yang A.L., Lin Y.M., Wu F.N., Lin J.A., Chan Y.S., Tsai F.J., Tsai C.H., Kuo C.H., Lee S.D. (2012). Anti-apoptotic and pro-survival effects of exercise training on hypertensive hearts. J. Appl. Physiol..

[9] Narula J., Haider N., Arbustini E., Chandrashekhar Y. (2006). Mechanisms of disease: Apoptosis in heart failure—Seeing hope in death. Nat. Clin. Pract. Cardiovasc. Med..

[10] Bennett M.R. (2002). Apoptosis in the cardiovascular system. Heart.

[11] Brown D.A., Perry J.B., Allen M.E., Sabbah H.N., Stauffer B.L., Shaikh S.R., Cleland J.G., Colucci W.S., Butler J., Voors A.A. (2017). Expert consensus document: Mitochondrial function as a therapeutic target in heart failure. Nat. Rev. Cardiol..

[12] Zhou Y., Wang S., Li Y., Yu S., Zhao Y. (2017). SIRT1/PGC-1alpha Signaling Promotes Mitochondrial Functional Recovery and Reduces Apoptosis after Intracerebral Hemorrhage in Rats. Front. Mol. Neurosci..

[13] Forni V., Wuerzner G., Pruijm M., Burnier M. (2011). Long-term use and tolerability of irbesartan for control of hypertension. Integr. Blood Press Control.

[14] Riveiro A., Mosquera A., Alonso M., Calvo C. (2002). Angiotensin II type 1 receptor blocker irbesartan ameliorates vascular function in spontaneously hypertensive rats regardless of oestrogen status. J. Hypertens..

[15] Zhang Z.Z., Shang Q.H., Jin H.Y., Song B., Oudit G.Y., Lu L., Zhou T., Xu Y.L., Gao P.J., Zhu D.L. (2013). Cardiac protective effects of irbesartan via the PPAR-gamma signaling pathway in angiotensin-converting enzyme 2-deficient mice. J. Transl. Med..

[16] Bramlage P., Pittrow D., Kirch W. (2004). The effect of irbesartan in reducing cardiovascular risk in hypertensive type 2 diabetic patients: An observational study in 16,600 patients in primary care. Curr. Med. Res. Opin..

[17] Kintscher U., Bramlage P., Paar W.D., Thoenes M., Unger T. (2007). Irbesartan for the treatment of hypertension in patients with the metabolic syndrome: A sub analysis of the Treat to Target post authorization survey. Prospective observational, two armed study in 14,200 patients. Cardiovasc. Diabetol..

[18] Watanabe R., Suzuki J., Wakayama K., Kumagai H., Ikeda Y., Akazawa H., Komuro I., Isobe M. (2016). Angiotensin II receptor blocker irbesartan attenuates cardiac dysfunction induced by myocardial infarction in the presence of renal failure. Hypertens. Res..

[19] Pai P.Y., Lin Y.Y., Yu S.H., Lin C.Y., Liou Y.F., Wu X.B., Wong J.K.S., Huang C.Y., Lee S.D. (2021). Angiotensin II receptor blocker irbesartan attenuates sleep apnea-induced cardiac apoptosis and enhances cardiac survival and Sirtuin 1 upregulation. Sleep Breath.

[20] Brunner H.R. (1997). The new angiotensin II receptor antagonist, irbesartan: Pharmacokinetic and pharmacodynamic considerations. Am. J. Hypertens..

[21] Fujino M., Miura S., Kiya Y., Tominaga Y., Matsuo Y., Karnik S.S., Saku K. (2010). A small difference in the molecular structure of angiotensin II receptor blockers induces AT(1) receptor-dependent and -independent beneficial effects. Hypertens. Res. Off. J. Jpn. Soc. Hypertens..

[22] Zhao Y., Watanabe A., Zhao S., Kobayashi T., Fukao K., Tanaka Y., Nakano T., Yoshida T., Takemoto H., Tamaki N. (2014). Suppressive effects of irbesartan on inflammation and apoptosis in atherosclerotic plaques of apoE-/- mice: Molecular imaging with 14C-FDG and 99mTc-annexin A5. PLoS ONE.

[23] Chang R.L., Nithiyanantham S., Huang C.Y., Pai P.Y., Chang T.T., Hu L.C., Chen R.J., VijayaPadma V., Kuo W.W., Huang C.Y. (2019). Synergistic cardiac pathological hypertrophy induced by high-salt diet in IGF-IIRalpha cardiac-specific transgenic rats. PLoS ONE.

[24] Finck B.N., Kelly D.P. (2007). Peroxisome proliferator-activated receptor gamma coactivator-1 (PGC-1) regulatory cascade in cardiac physiology and disease. Circulation.

[25] Lehman J.J., Barger P.M., Kovacs A., Saffitz J.E., Medeiros D.M., Kelly D.P. (2000). Peroxisome proliferator-activated receptor gamma coactivator-1 promotes cardiac mitochondrial biogenesis. J. Clin. Investig..

[26] Karamanlidis G., Garcia-Menendez L., Kolwicz S.C., Lee C.F., Tian R. (2014). Promoting PGC-1alpha-driven mitochondrial biogenesis is detrimental in pressure-overloaded mouse hearts. Am. J. Physiol. Heart Circ. Physiol..

[27] Sihag S., Cresci S., Li A.Y., Sucharov C.C., Lehman J.J. (2009). PGC-1alpha and ERRalpha target gene downregulation is a signature of the failing human heart. J. Mol. Cell Cardiol..

[28] Lin Y.Y., Hong Y., Yu S.H., Wu X.B., Shyu W.C., Chen J.S., Ting H., Yang A.L., Lee S.D. (2019). Antiapoptotic and mitochondrial biogenetic effects of exercise training on ovariectomized hypertensive rat hearts. J. Appl. Physiol..

[29] Kusunoki H., Taniyama Y., Rakugi H., Morishita R. (2013). Cardiac and renal protective effects of irbesartan via peroxisome proliferator-activated receptorgamma-hepatocyte growth factor pathway independent of angiotensin II Type 1a receptor blockade in mouse model of salt-sensitive hypertension. J. Am. Heart Assoc..

[30] Matsushima S., Sadoshima J. (2015). The role of sirtuins in cardiac disease. Am. J. Physiol. Heart Circ. Physiol..

[31] Alcendor R.R., Gao S., Zhai P., Zablocki D., Holle E., Yu X., Tian B., Wagner T., Vatner S.F., Sadoshima J. (2007). Sirt1 regulates aging and resistance to oxidative stress in the heart. Circ. Res..

[32] Elibol B., Kilic U. (2018). High Levels of SIRT1 Expression as a Protective Mechanism Against Disease-Related Conditions. Front. Endocrinol..

[33] Ren Y., Sun C., Sun Y., Tan H., Wu Y., Cui B., Wu Z. (2009). PPAR gamma protects cardiomyocytes against oxidative stress and apoptosis via Bcl-2 upregulation. Vascul. Pharmacol..

[34] Archer S.L. (2013). Mitochondrial dynamics--mitochondrial fission and fusion in human diseases. N. Engl. J. Med..

[35] Quiles J.M., Gustafsson A.B. (2022). The role of mitochondrial fission in cardiovascular health and disease. Nat. Rev. Cardiol..

